# IGFBP7 Fuels the Glycolytic Metabolism in B-Cell Precursor Acute Lymphoblastic Leukemia by Sustaining Activation of the IGF1R–Akt–GLUT1 Axis

**DOI:** 10.3390/ijms24119679

**Published:** 2023-06-02

**Authors:** Leonardo Luís Artico, Juliana Silveira Ruas, José Ricardo Teixeira Júnior, Natacha Azussa Migita, Gustavo Seguchi, Xinghua Shi, Silvia Regina Brandalise, Roger Frigério Castilho, José Andrés Yunes

**Affiliations:** 1 Centro Infantil Boldrini, Campinas 13083-210, SP, Brazil; lla.unicamp2017@gmail.com (L.L.A.); ruasjulianas@gmail.com (J.S.R.); jrteixeira094@gmail.com (J.R.T.J.); azussamigita@gmail.com (N.A.M.); gseguchi@gmail.com (G.S.); silvia@boldrini.org.br (S.R.B.); 2 Graduate Program in Genetics and Molecular Biology, Institute of Biology, University of Campinas, Campinas 13083-862, SP, Brazil; 3 Department of Computer and Information Sciences, Temple University, Philadelphia, PA 19122, USA; mindyshi@temple.edu; 4 Department of Pathology, School of Medical Sciences, University of Campinas, Campinas 13083-887, SP, Brazil; rogerc@unicamp.br

**Keywords:** IGFBP7, IGF1R, GLUT1, PI3K–Akt, Acute Lymphoblastic Leukemia

## Abstract

Increased glycolytic metabolism plays an important role in B-cell precursor Acute Lymphoblastic Leukemia (BCP-ALL). We previously showed that IGFBP7 exerts mitogenic and prosuvival effects in ALL by promoting IGF1 receptor (IGF1R) permanence on the cell surface, thus prolonging Akt activation upon IGFs/insulin stimulation. Here, we show that sustained activation of the IGF1R–PI3K–Akt axis concurs with GLUT1 upregulation, which enhances energy metabolism and increases glycolytic metabolism in BCP-ALL. IGFBP7 neutralization with a monoclonal antibody or the pharmacological inhibition of the PI3K–Akt pathway was shown to abrogate this effect, restoring the physiological levels of GLUT1 on the cell surface. The metabolic effect described here may offer an additional mechanistic explanation for the strong negative impact seen in ALL cells in vitro and in vivo after the knockdown or antibody neutralization of IGFBP7, while reinforcing the notion that it is a valid target for future therapeutic interventions.

## 1. Introduction

Acute Lymphoblastic Leukemia (ALL) is the most common childhood cancer. Although survival rates for pediatric ALL have reached around 90% in the last decade, 20% to 30% of patients still relapse [1,2,3], and some experience long-term sequelae of therapy including a second malignancy [4,5,6]. Therefore, new drugs and therapeutic strategies are needed.

Fine-tuning of the glycolytic pathway is a critical step in the malignant transformation of B-cells [7,8]. In B-cell precursor Acute Lymphoblastic Leukemia (BCP-ALL), increased glycolytic metabolism plays an important role in ALL [9,10]. In immune cells, including BCP-ALL, Glucose Transporter 1 (GLUT1) coordinates glucose uptake [10,11]. Transcriptional regulation, increased plasma membrane targeting, and the recycling of GLUT1 are directly regulated by insulin, growth factors, and cytokines [12,13]. It is well-established that in leukemia cells, activation of the PI3K–Akt axis by receptor tyrosine kinases or oncogenic lesions (gain-of-function mutations) induces GLUT1 accumulation at the cell surface and increases aerobic glycolysis [14]. Recently, a feedforward loop between glycolytic ATP production and PI3K–Akt pathway activation was reported in T-cells [15,16], highlighting the importance of this crosstalk for cellular activation and survival. Together, these observations point to an important role for PI3K–Akt in the control of glucose metabolism in immune cells and in the Warburg effect on blood cell disorders, such as BCP-ALL.

We previously showed that IGFBP7 (insulin-like growth factor-binding protein 7) exerts mitogenic and prosurvival autocrine effects in ALL [17]. Under insulin/recombinant insulin-like grow factor 1 (rIGF1) stimulation, recombinant IGFBP7 (rIGFBP7) promotes IGF1 receptor (IGF1R) permanence on the cell surface, fueling the PI3K–Akt pathway and, consequently, increasing cell viability/proliferation. We also demonstrated that IGFBP7 is a valid target for antibody-based therapeutic interventions in ALL [17]. Although the positive regulatory circuitry between PI3K–Akt and glycolytic metabolism is crucial for immune cells’ survival, almost nothing is known about the existence of extracellular proteins (e.g., IGFBP7) that are possibly regulating this system, which may also be important in the oncogenic context, including ALL. Here, we show that the rIGFBP7-mediated rIGF1/IGF1R sustained activation of the PI3K–Akt pathway concurs with GLUT1 upregulation and increased glycolytic metabolism in BCP-ALL.

## 2. Results and Discussion

We previously showed that the treatment of ALL cells with insulin+rIGFBP7 prolongs IGF1R signaling (but not the insulin receptor, INSR) and Akt phosphorylation for up to 4 h [17]. Here, we confirmed this finding in a timecourse experiment. Treatment of BCP-ALL cell lines (RS4;11 and 697) with rIGF1+rIGFBP7 resulted in IGF1R signaling and Akt phosphorylation that reached a peak in about 15 min and lasted for 4 h, while phosphorylation of the INSR, after peaking at 15 min, rapidly decreased to low levels in 1 h (Figure S1A). These data indicate that IGFBP7 effects are not restricted to merely extending the half-life of the IGF1 ligand, since in that case both IGF1R and INSR should have been activated at the 4 h point. Overall, these findings confirm that IGF1R is the primary mediator of the IGFBP7 enhancement of IGF1 activity in BCP-ALL.

**Figure 1:**
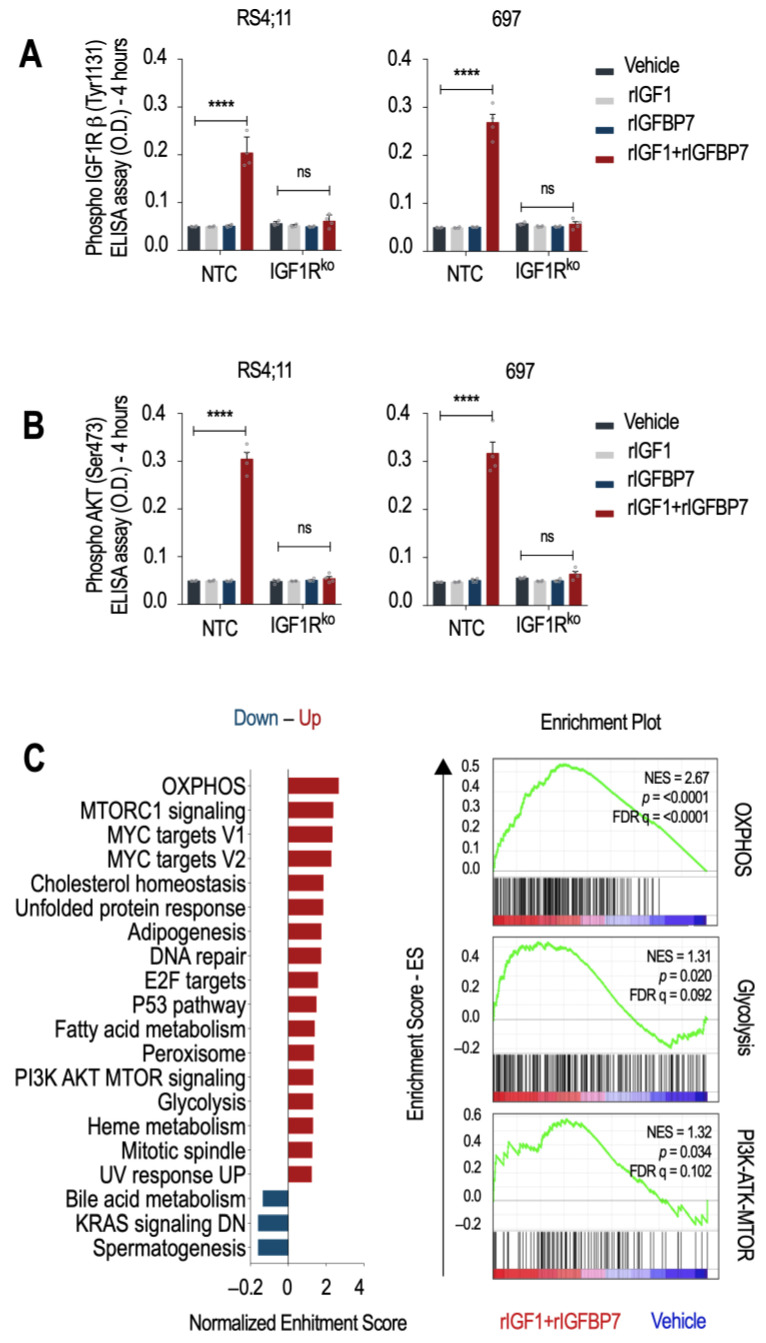
IGFBP7 sustains activation of the IGF1R/Akt axis under IGF1 stimulation in an IGF1R-dependent manner in BCP-ALL cells. (**A**,**B**) ELISA results for (**A**) phospho-IGF1Rβ (Tyr1131) and (**B**) phospho-Akt (Ser473) in RS4;11 and 697 BCP-ALL cell lines (no target control (NTC) and IGF1R knockout) after 4 h of treatment with rIGF1 (50 ng/mL) and/or rIGFBP7 (100 ng/mL). Bars represent means ± SEM for four independent experiments; ns = not significant. (**C**) Left: GSEA analysis results. Statistically significant hallmark gene sets were selected (FDR < 0.25) and distributed by the Normalized Enrichment Score (NES). Red bars indicate upregulated hallmark gene sets in BCP-ALL cell lines after 6 h stimulation with rIGF1+rIGFBP7 (50 ng/mL and 100 ng/mL, respectively) in serum-free medium. Blue bars indicate downregulated hallmark gene sets. Right: GSEA enrichment plots for selected hallmarks. Green curve corresponds to the enrichment score (ES). The normalized enrichment score (NES) represents the strength of the relationship between phenotype and gene signature. Bars represent means ± SEM for three independent experiments. Statistical analyses were completed by 2-way ANOVA and Bonferroni post-tests (**** *p* ≤ 0.0001).

To test whether IGF1R is indispensable for the transduction of the rIGF1+rIGFBP7 stimulus to downstream effectors such as Akt, we generated two IGF1R knockout cell lines (IGF1Rko—Figure S1B,C). As expected, NTC cells (no target control) maintained detectable levels of IGF1R and Akt phosphorylation (but not INSR) 4 h after rIGF1+rIGFBP7 co-treatment, while no such sustained signaling was seen in IGF1Rko cells nor when rIGF1 or rIGFBP7 were used separately (Figure 1A,B and Figure S1D). A previous study demonstrated that the N-terminal 97 amino acid fragment of IGFBP7 binds to the extracellular domain of IGF1R and prevents its internalization in response to IGF1 [18]. In our previous study, we confirmed this finding by showing that IGFBP7 prevented the internalization of IGF1R in ALL cells after insulin/IGF1 stimulation [17]. Together, these results support the idea that IGFBP7 functions by binding to and stabilizing the IGF1R receptor on the surface of ALL cells, thereby extending its response to IGF1 or insulin. The mechanism responsible for IGF1R retention at the cell surface is not yet known and deserves further investigation.

To better understand the cellular consequences of prolonged IGF1R/Akt signaling in BCP-ALL, we performed a microarray gene expression analysis followed by a Gene Set Enrichment Analysis (GSEA) [19,20]. Hallmark gene sets characteristic of cell growth (PI3K/Akt/mTOR, mTORC1, MYC targets, E2F targets, and mitotic spindle) and metabolism (oxidative phosphorylation-OXPHOS, adipogenesis, fatty acid metabolism, and glycolysis) were upregulated upon rIGF1+rIGFBP7 treatment of BCP-ALL cell lines (Figure 1C). This result is in agreement with reports showing that INSR and IGF1R deliver overlapping transcriptional responses, even though, at the organism level, insulin acts on metabolism, and IGF1 acts on growth [21]. As we know, dysregulation of these pathways was linked to the development and progression of several cancers, including ALL [22]. In general, activation of the PI3K/Akt/mTOR pathway downstream of IGF signaling promotes cell survival, proliferation, and growth by regulating protein translation, ribosome biogenesis, and cell cycle progression [23], which is in agreement with our previous findings on ALL [17]. Insulin and IGFs signaling also impact cellular metabolism by regulating OXPHOS, adipogenesis, fatty acid metabolism, and glycolysis. Activation of these pathways leads to increased glucose uptake and utilization, lipid synthesis, and mitochondrial biogenesis, ultimately supporting cell growth and proliferation [24]. As IGFBP7 enhances the signaling of these pathways, it might also contribute to the glycolytic metabolism of BCP-ALL.

**Figure 2:**
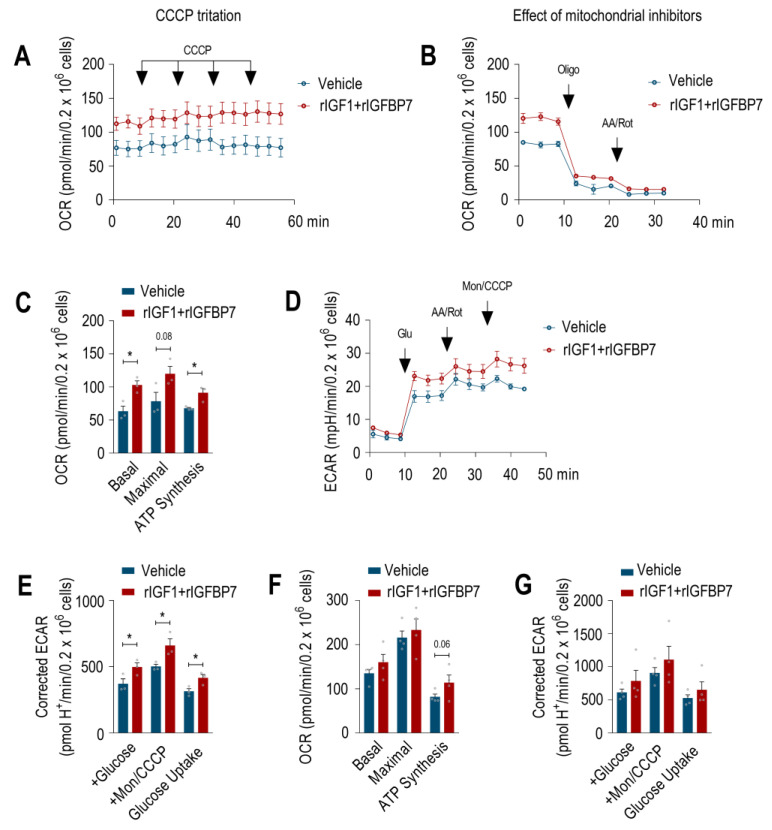
IGFBP7 enhances the energy metabolic parameters of BCP-ALL cells after IGF1 stimulation. Oxygen consumption rate (OCR) traces in 697 and RS4;11 cell lines after 4 h of treatment with rIGF1+rIGFBP7 (50 ng/mL and 100 ng/mL, respectively) or control (vehicle). (**A**) Arrows indicate sequential injections of CCCP (total amount of 1.2 µM) to reach maximal OCR in 697 cells. (**B**) Arrows indicate oligomycin (Oligo, 1 μg/mL) and antimycin+rotenone (AA/Rot, 1 μM each) injections to evaluate fractions of OCR linked to ATP synthesis and non-mitochondrial OCR, respectively, in 697 cells. (**C**) OCR rates (basal, maximal, and linked to ATP synthesis) in 697 cell line. Bars or curves represent means ± SEM for three independent experiments. (**D**) Representative extracellular acidification rate (ECAR) traces in 697 cell line after 4 h of treatment with rIGF1+rIGFBP7 (50 ng/mL and 100 ng/mL, respectively) or control (vehicle). Arrows indicate glucose (Glu, 10 mM), antimycin+rotenone (AA/Rot, 1 μM each) and monensin+CCCP (Mon, 200 μM; CCCP, 1 μM) injections. (**E**) Individual ECAR rates in 697 cell line (basal (+glucose), maximal (Mon+CCCP), and glucose uptake (basal with glucose—basal without glucose)). Bars represent means ± SEM for three independent experiments. (**F**) OCR rates (basal, maximal, and linked to ATP synthesis) in the RS4;11 cell line. Bars or curves represent means ± SEM for four independent experiments. (**G**) Individual ECAR rates in the RS4;11 cell line (basal (+glucose), maximal (Mon+CCCP), and glucose uptake (basal with glucose—basal without glucose)). Bars represent means ± SEM for four independent experiments. Statistical analyses were completed by unpaired *t*-test (* *p* ≤ 0.05).

In order to check whether IGFBP7 contributes to the improvement of energy metabolism on BCP-ALL, we determined the oxygen consumption rate (OCR) and extracellular acidification rate (ECAR) in BCP-ALL cells upon rIGF1+rIGFBP7 stimulation to assess OXPHOS and glycolysis, respectively. Both the basal and maximal OCR of the 697 cell line increased upon treatment, along with the OCR coupled to ATP synthesis (Figure 2A–C). In addition, when mitochondrial OXPHOS was chemically inhibited, treated 697 cells showed significantly increased glycolytic metabolism (Figure 2D,E). A similar trend toward increased OCR and ECAR following rIGF1+rIGFBP7 treatment was found in the RS4;11 ALL cell line, even though the differences were non-significant at the statistical level (Figure 2F,G and Figure S2).

**Figure 3:**
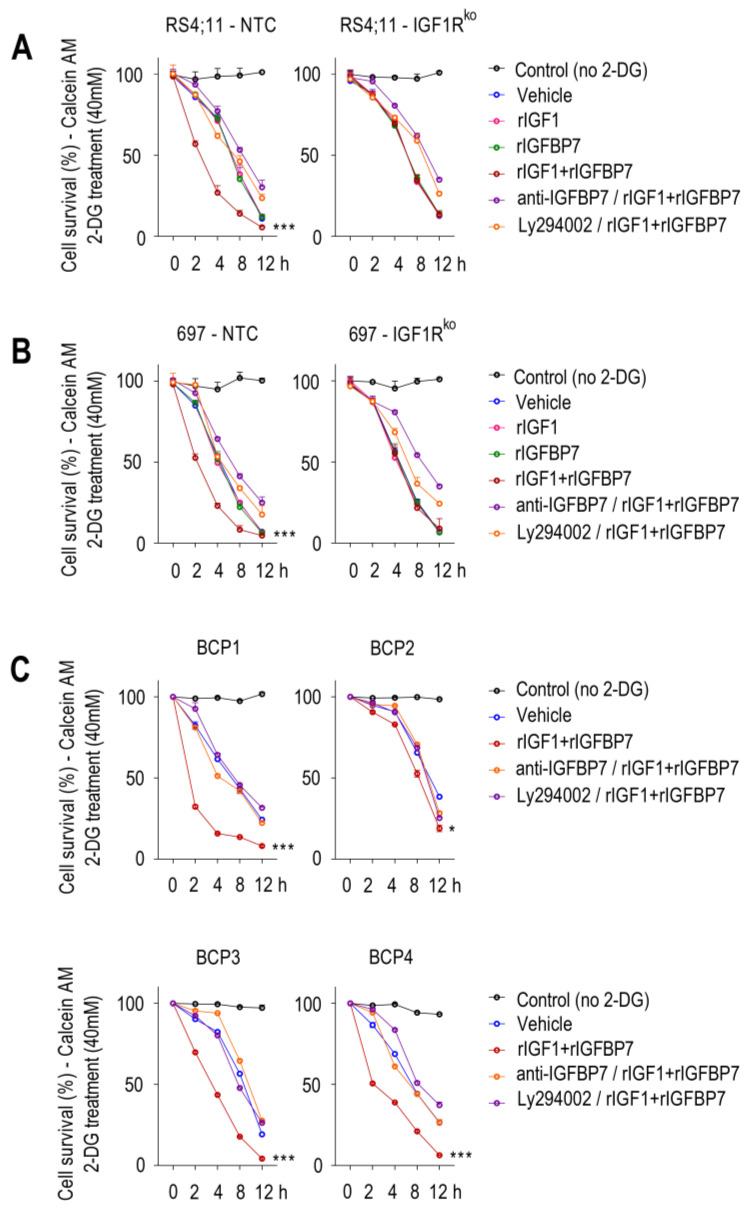
IGFBP7 potentiates glucose uptake in BCP-ALL in an IGF1-, IGF1R-, and PI3K-dependent manner, as evaluated by the 2-DG induced cell death assay. (**A**–**C**) Calcein AM cell viability assay in (**A**) RS4;11 and (**B**) 697 BCP-ALL cell lines (no target control (NTC) and IGF1R knockout) and (**C**) primary BCP-ALL cells. Except for the control (no 2-DG; black lines), in all other conditions cells were pretreated with rIGF1 (50 ng/mL) and/or rIGFBP7 (100 ng/mL) for 4 h and then subjected to 2-DG treatment (40 mM) for up to 12 h. Where indicated, cells were pretreated with an anti-IGFBP7 antibody (clone C311, 20 µg/mL) or Ly294002 (30 µM), which was added 30 min before rIGF1+rIGFBP7 treatment initiation. Viable cells (Calcein +) were measured by flow cytometry at each indicated time point. Curves represent means ± SEM for three independent experiments. Statistical analyses correspond to differences between areas under the curves (AUC) (* *p* ≤ 0.05; *** *p* ≤ 0.001).

Since Akt activation induces aerobic glycolysis by promoting glucose uptake by the cell [25,26], we tested if the increased glycolytic capacity of rIGF1+rIGFBP7-treated BCP-ALL cells would rely on Akt activation and increased glucose uptake as well. To assess glucose uptake, we cultured cells in medium supplemented with 2-deoxy-D-glucose (2-DG), a glucose analog that induces cell death at high concentrations. In this assay, cell death would indicate higher 2-DG uptake. Sensitivity to lethal 2-DG supplementation was clearly more pronounced when cells were pretreated with rIGF1+rIGFBP7 than by any of these factors individually (Figure 3A,B). No such additive effect was seen with the IGF1Rko cells, indicating that IGF1R was indispensable for the increased glucose uptake triggered by rIGF1+rIGFBP7. Likewise, rIGFBP7 neutralization with an anti-IGFBP7 monoclonal antibody [17] or PI3K inhibition with Ly294002 protected rIGF1+rIGFBP7-stimulated BCP-ALL cells from the lethal effects of 2-DG, highlighting the importance of these two components (IGFBP7 and PI3K) in glucose uptake regulation in BCP-ALL. Similar results were observed in four primary BCP-ALL cells (Table S1 and Figure 3C). The level of sustained Akt phosphorylation upon rIGF1+rIGFBP7 treatment correlated to the degree of 2-DG sensitivity in BCP-ALL cell lines (Figure S3) and in primary BCP-ALL cells (Figure S4). Importantly, similar results were found when rIGF1 was substituted by insulin (Figure S5).

**Figure 4:**
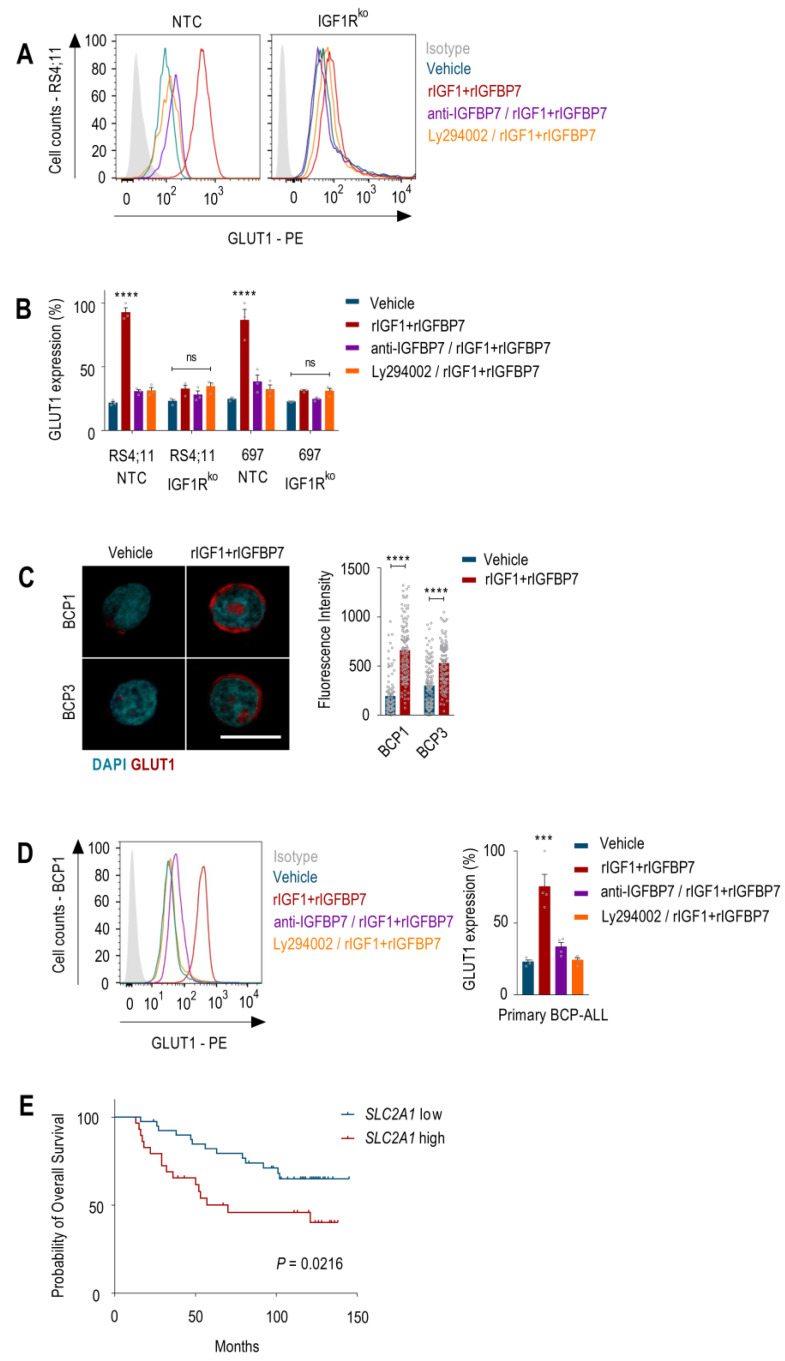
IGFBP7 promotes GLUT1 expression at the cell surface of BCP-ALL in an IGF1-, IGF1R-, and PI3K-dependent manner. (**A**) GLUT1 cell surface expression obtained by flow cytometry in the RS4;11 and 697 BCP-ALL cell lines (no target control (NTC) and IGF1R knockout) after 24 h of treatment with rIGF1+rIGFBP7 (50 ng/mL and 100 ng/mL, respectively) or control (vehicle). Where indicated, cells were pretreated with an anti-IGFBP7 antibody (clone C311, 20 µg/mL) or Ly294002 (30 µM), which was added 30 min before rIGF1+rIGFBP7. (**B**) Normalized results of GLUT1 surface expression from three independent experiments. Bars represent means ± SEM; ns = not significant. (**C**) Confocal analysis of GLUT1 expression in two primary BCP-ALL (BCP1 and 3) samples collected 24 h after treatment with rIGF1+rIGFBP7 (50 ng/mL and 100 ng/mL, respectively) or control (vehicle). Scale bar: 10 μm. Bars represent means ± SEM of the fluorescence intensity of individual cells (symbols). (**D**) GLUT1 cell surface expression obtained by flow cytometry in primary BCP-ALL cells treated for 24 h with rIGF1+rIGFBP7 (50 ng/mL and 100 ng/mL, respectively) or control (vehicle). Where indicated, cells were pretreated with an anti-IGFBP7 antibody (clone C311, 20 µg/mL) or Ly294002 (30 µM), which was added 30 min before rIGF1+rIGFBP7. Left: GLUT1 cell surface expression in a representative case (BCP1). Right: normalized results for GLUT1 cell surface expression for four different primary BCP-ALL samples. (**E**) Kaplan–Meier survival curves generated by the cBioPortal (http://www.cbioportal.org (accessed on 15 February 2023)). Overall survival curves of BCP-ALL patients according to low (*n* = 40) versus high (*n* = 41) *SLC2A1* expression. Curves were compared by the log-rank test. Bars represent means ± SEM. Differences were compared by 1- or 2-way ANOVA and Bonferroni post-tests (*** *p* ≤ 0.001; **** *p* ≤ 0.0001).

Studies showed that Akt activation results in increased amounts of GLUT1 at the plasma membrane of both normal and malignant hematopoietic cells [25,27,28]. Chronic Akt activation was shown to stabilize GLUT1 at the cell surface by inhibiting the endocytic machinery [29,30]. Accordingly, 24 h after rIGF1+rIGFBP7 treatment, BCP-ALL cells showed ~60% more GLUT1 on their cell surface than untreated cells. As anticipated, this effect was completely abrogated when the cells were pretreated with anti-IGFBP7 antibody or Ly294002. Moreover, IGF1Rko cells showed no changes in GLUT1 surface expression under any tested condition (Figure 4A,B). In addition, compatible results were found in primary BCP-ALL samples (Figure 4C,D). GLUT1 overexpression (*SLC2A1*) had a negative prognostic impact on several types of cancer [12], including BCP-ALL (Figure 4E). The glucose metabolism was implicated in the mechanism of action and resistance to L-asparaginase [31] and glucocorticoids [32,33,34] in ALL. Therefore, understanding the molecular mechanism that governs GLUT1 expression and glycolysis in ALL seems to be extremely important.

Activation of the PI3K–Akt pathway in immune cells triggers the glycolytic metabolism, primarily by promoting GLUT1 expression and the transport of glucose across the plasma membrane [7,8,9,10]. Numerous studies suggested that targeting the PI3K–Akt pathway could be a potential approach to disrupt GLUT1 activity and block glycolytic metabolism in ALL, thereby contributing to disease eradication [25,27,28,29,30].

Despite a wealth of knowledge about the pro-survival effects mediated by the PI3K–Akt pathway in cancer, the non-classical physiological mechanisms regulating this pathway remain poorly understood. Previously, we demonstrated a significant activation of the insulin/IGF1–IGF1R–PI3K–Akt axis by IGFBP7 in ALL. Our data showed that the physiological levels of IGFBP7 sustain the surface expression and activation of IGF1R by insulin/IGF1, revealing a new mechanism of ALL survival and chemotherapy resistance [17,35]. We also demonstrated that the genetic knockdown or IGFBP7 neutralization using an anti-IGFBP7 monoclonal antibody significantly decreased ALL progression in vivo, highlighting IGFBP7 as a potential therapeutic target for ALL [17].

**Figure 5:**
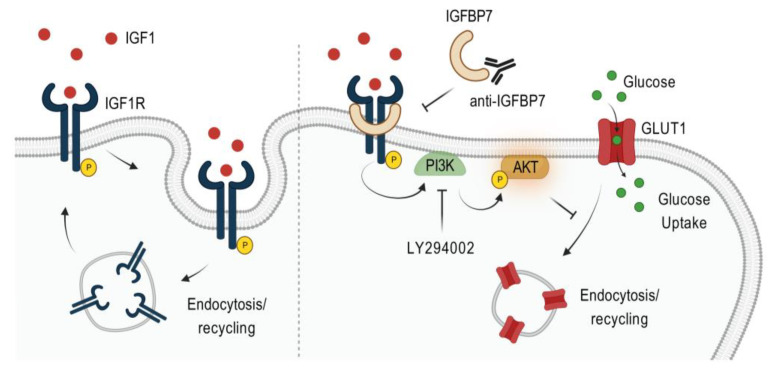
Schematic diagram illustrating the proposed mechanism by which IGFBP7 stimulates the glycolytic metabolism of ALL. In presence of IGFBP7, IGF1R remains active on the cell surface and sustains the activation of the PI3K–Akt pathway for a longer time. Active Akt blocks GLUT1 endocytosis/recycling, thus resulting in more GLUT1 at the cell surface and in increased glucose transport to fuel the glycolytic metabolism. IGFBP7 neutralization with a monoclonal antibody or the pharmacological inhibition of PI3K–Akt pathway was shown to abrogate this effect, restoring the physiological levels of GLUT1 on the cell surface. The mechanism by which IGFBP7 retains IGF1R at the cell surface is not known. Figure created with BioRender.com.

Although we extensively explored and discussed the molecular mechanism underlying these effects in our previous studies [17,35], here, we present a novel avenue for therapeutic interventions targeting the glycolytic metabolism in ALL. Our findings support the involvement of IGFBP7 in GLUT1 expression and in promoting an increased glycolytic metabolism in BCP-ALL. Specifically, we discovered that the rIGF1+rIGFBP7-mediated activation of the PI3K–Akt pathway was mainly dependent on IGF1R, as knocking out this receptor in the BCP-ALL cell lines resulted in signaling abrogation. To confirm the role of both IGFBP7 and PI3K–Akt in enhancing the glycolytic activity of BCP-ALL, we employed two strategies: (1) neutralization of the IGFBP7 protein with a monoclonal antibody and (2) inhibition of PI3K with the Ly294002 drug. Both approaches resulted in significant inhibition of the sustained PI3K–Akt–GLUT1 signaling induced by rIGF1+rIGFBP7, thus validating the significance of the IGF1+IGFBP7–IGF1R–Akt–GLUT1 axis in regulating the glycolytic metabolism of BCP-ALL (Figure 5).

The metabolic effect described here may offer an additional mechanistic explanation for the strong negative impact seen in ALL cells in vitro and in vivo after the knockdown or antibody neutralization of IGFBP7 [17], while reinforcing the notion that it is a valid target for future therapeutic interventions. In conclusion, our observations reveal a hitherto unknown role for IGFBP7 in the glycolytic metabolism of BCP-ALL, opening doors for future investigations.

## 3. Materials and Methods

### 3.1. Cell Culture

BCP-ALL cell lines and primary BCP-ALL were cultured as described by Artico et al. [17]. The use of primary BCP-ALL for in vitro experiments was approved by the Research Ethics Committee from the State University of Campinas (CAAE: 0014.0.144.146-08 and 0018.0.144.146-08).

### 3.2. Knockout of IGF1R in BCP-ALL Cell Lines

Knockout of IGF1R in RS4;11 and 697 cells was performed using the all-in-one doxycycline inducible CRISPR system with lentiviral vector TLCV2 (Ref. #87360, Addgene, Watertown, MA, USA). We designed a guide sequence to knockout IGF1R at exon 3 (IGF1R gRNA: 5′-CACCGGCATAGTAGTAGTGG-3′) and a non-targeting sequence as a control (NTC gRNA: 5′-AAATGTGAGATCAGAGTAAT-3′). Guide-sequence oligonucleotides were phosphorylated with T4 PNK (Thermo Fisher Scientific, Waltham, WA, USA) and annealed by incubation in a thermocycler under the following conditions: 30 min at 37 °C, 5 min at 95 °C, ramp down to 25 °C at 5 °C per min. TLCV2 was digested with Esp3I (Thermo Fischer Scientific) and ligated to the phosphorylated and annealed oligonucleotides by incubation at 22 °C for 1 h with T4 DNA ligase. Competent Stbl3 *E. coli* cells were transformed with each ligation reaction, and plasmid DNA was extracted with the NucleoSpin Plasmid Mini kit (Macherey-Nagel, Düren, Germany). For production of lentiviral particles containing each TLCV2 construct, we used the second-generation lentiviral package plasmid psPAX2 (Ref. #12260, Addgene, Watertown, MA, USA and the VSV-G envelope plasmid pMD2.G (Ref. #12259, Addgene, Watertown, MA, USA). HEK293T cells cultured in DMEM medium containing 10% FBS and 1% penicillin/streptomycin were transfected at 70% confluence with each TLCV2 construct and helper plasmids at a ratio of 3:2:1 TLCV2:psPAX2:pMD2.G, with 1 µg of DNA per mL of culture medium. Transfection was performed using polyethylenimine (PEI; Sigma-Aldrich, St. Louis, MO, USA) at a ratio of 3:1 PEI:DNA (*w*/*w*), with both PEI and DNA diluted in Opti-MEM (Thermo Fisher Scientific, Waltham, MA, USA) at a volume 1/10 of the total volume of culture medium, followed by incubation for 20 min before being applied to the cells. Transfection medium was replaced with culture medium without antibiotics 16 h after transfection. Supernatant containing lentiviral particles was collected 24 h and 48 h later and filtered through a 0.45 µm PVDF membrane. RS4;11 and 697 cells were transduced by spinfection with Polybrene (Sigma-Aldrich, St. Louis, MO, USA). Polybrene was applied to the supernatant containing lentiviral particles to a final concentration of 8 µg/mL. Next, the supernatant was used to resuspend 3.0 × 10^5^ cells in a 24-well plate, which was then centrifuged at 800× *g* for 1 h at room temperature. Transduction medium was replaced with culture medium 6 h after transduction. Two days later, puromycin (Thermo Fisher Scientific, Waltham, MA, USA) was added to the medium at a concentration of 2 µg/mL to select transduced cells. To induce Cas9 expression from the TLCV2 construct, cells were incubated with 1 µg/mL doxycycline (Sigma-Aldrich, St. Louis, MO, USA) for 48 h. Cells were then incubated with anti-IGF1R (anti-hCD221-BV421, clone 1H7) and/or anti-INSR (anti-hCD220-PE, clone 3B6) antibodies (Becton Dickinson, Franklin Lakes, NJ, USA) or the corresponding isotype controls (mIgG1k-BV421, clone X40 and mIgG1k-PE, clone MOPC-21, respectively; Becton Dickinson, Franklin Lakes, NJ, USA) and then analyzed by flow cytometry. Cells transduced with the TLCV2-IGF1R sgRNA construct that were positive for EGFP (indicating induction of Cas9 expression) and negative for surface IGF1R (relative to cells transduced with the TLCV2-NTC sgRNA) were sorted with BD FACSAriaTM Fusion (Becton Dickinson, Franklin Lakes, NJ, USA).

### 3.3. Enzyme-Linked Immunosorbent Assay—ELISA

BCP-ALL cells (1 × 10^6^) were incubated for 4 h in serum-free medium (RPMI1640 for cell lines and AIM-V for primary cells). Afterwards, cells were treated with rIGF1 (50 ng/mL, R&D Systems Biotechnology, Minneapolis, MI, USA) and/or rIGFBP7 (100 ng/mL, R&D Systems Biotechnology, Minneapolis, MI, USA) for 4 h. Where indicated, cells were pretreated with an anti-IGFBP7 antibody (clone C311, 20 µg/mL) [17] or Ly294002 (30 µM, #9901 Cell Signaling Technology, Danvers, MA, USA), which was added 30 min before rIGF1+rIGFBP7 treatment initiation. Enzyme-linked immunosorbent assay (ELISA) for phospho-IGF1R, phospho-INSR, and phospho-Akt in BCP-ALL cell lines and primary BCP-ALL cells was performed as described by Artico et al. [17].

### 3.4. Differential Expression Array Analysis

Gene expression analysis were performed using three different BCP-ALL cell lines (697, RS4;11, and REH), untransformed or transformed with lentiviral MISSION pLKO.1-puro shRNA expression vectors (Sigma-Aldrich, St. Louis, MO, USA) against IGFBP7 (shRNA #812 and shRNA #959) or non-template control (shRNA Scrambled) [11]. The experiments were performed in 6-well plates (1 × 10^6^ cells/well), with all four versions of each of the cell lines (WT, Scrambled, #812, and #959). Cells were starved during 4 h in serum-free RPMI1640 medium, and then two wells for each version were stimulated with rIGF1+rIGFBP7 (50 ng/mL and 100 ng/mL, respectively) and two wells with vehicle (control, PBS) for 6 h. Cells from the duplicate wells were joined, and total RNA was extracted using the Illustra RNA Mini Spin kit (GE Healthcare Life Sciences, Chicago, IL, USA). RNA was quantified using Qubit Fluorometer (Thermo Fisher Scientific, Waltham, MA, USA) and run in agarose gel electrophoresis for quality assessment. The isolated RNA was amplified, labeled, and purified to obtain biotin-labeled cRNA using the reagents and enzymes supplied in the GeneChip^®^ WT Pico Reagent Kit (Affymetrix, Termo Fisher Scientifc, Waltham, MA, USA), in accordance with the instructions of the manufacturer. Array hybridization and washes were performed using GeneChip^®^ Hybridization, Wash, and Stain kit (Affymetrix, Thermo Fisher Scientific, Waltham, MA, USA) on a Hybridization Oven 645 and a Fluidics Station 450 (Affymetrix, Thermo Fisher Scientific, Waltham, MA, USA). Slides were scanned using GeneChip^®^ Scanner 3000 (Affymetrix, Thermo Fisher Scientific, Waltham, MA, USA) and Command Console software 3.1 (Affymetrix, Thermo Fisher Scientific, Waltham, MA, USA) with default settings. CEL files were analyzed using Affymetrix Expression Console software (version 4.0.1). A Principal Component Analysis (PCA) in Affymetrix Expression Console software (version 4.0.1) revealed that the expression values for the RS4;11 #812 cells (both untreated or treated with rIGFBP7+rIGF1) were outliers and were, therefore, discarded from subsequent analysis. Gene expression values were obtained using the Signal Space Transformation-Robust Multi-Chip Analysis (SST-RMA) analysis algorithm. Log2 expression values were used for Gene Set Enrichment Analysis (GSEA) [19,20] against the Hallmark gene sets [36]. As differences between WT/Scrambled versus knockdown cell lines were not strong enough to overcome differences due to the genetic background of the cell lines used, we found it better to simply compare, irrespectively of any genetic modification, rIGF1+rIGFBP7-treated versus -untreated (vehicle). Untreated (vehicle) WT, Scrambled, #812, and #959 cells from the three different cell lines were all together in one group (*n* = 11, because RS4;11_#812 was excluded), whereas rIGF1+rIGFBP7-treated WT, Scrambled, #812, and #959 cells (*n* = 11) were in the other group. By doing so, we increased the sample size and the statistical discrimination of differences among groups under comparison. Significant Hallmark gene sets were selected (nominal *p* values ≤ 0.05) and distributed by the Normalized Enrichment Score (NES).

### 3.5. Extracellular Flux Analysis in 697 and RS4;11 Cell Lines

Oxygen consumption (OCR) and extracellular acidification rate (ECAR) in 697 and RS4;11 cell lines were measured using a Seahorse XF24 Analyzer (Agilent Technologies, Santa Clara, CA, USA). Cells were starved (serum-free) for 4 h and then treated with rIGF1+rIGFBP7 (50 ng/mL and 100 ng/mL, respectively) or vehicle (PBS) for 4 h. Cells were seeded at 2 × 10^5^/well in plates previously treated with poly-D-lysine (100 µg/mL, Sigma-Aldrich, St. Louis, MO, USA) and then submitted to centrifugation at 300× *g* for 5 min at room temperature to attach the cells to the well surface. Initially, 500 μL of RPMI1640 medium was placed in each well, and each injection consisted of 75 μL of the respective RPMI1640 medium with the target compound, resulting in a final volume of 800 μL. Three protocols were established to analyze oxidative phosphorylation (OXPHOS) and glycolysis. For OXPHOS, two experimental protocols were performed with RPMI1640 (pH 7.4), containing 10 mM glucose, 1 mM pyruvate, and 2 mM glutamine as metabolic substrates. The first protocol was intended to analyze basal oxygen consumption rate (OCR), maximal OCR, and spare respiratory capacity (SRC). Thus, all four injections were performed with protonophore carbonyl cyanide m-chlorophenylhydrazone (CCCP) to reach final concentrations in wells of 400 nM, 800 nM, 1000 nM, and 1200 nM. The second protocol was to evaluate the mitochondrial parameters such as the fraction of OCR linked to ATP synthesis, H+ leak, and non-mitochondrial OCR. The first addition consisted of 1 μg/mL oligomycin, and the second was of 1 μM antimycin A plus 1 μM rotenone. These two protocols were performed separately in order to avoid underestimation of parameters, maximal OCR and spare respiratory capacity, which can occur in the presence of oligomycin [37,38]. For glycolysis estimation, ECAR was measured, and the protocol was performed with RPMI1640 (pH 7.4) containing 2 mM glutamine. The first addition consisted of 10 mM glucose, the second of 1 μM antimycin A plus 1 μM rotenone, and the third of 200 μM monensin plus 1 μM CCCP [39]. ECAR values were corrected for non-glycolytic acidification [39], considering glutamine as the predominant mitochondrial metabolic substrate. Before the initiation of the three protocols described above, cells were maintained in incubator at 37 °C (without CO_2_) for 1 h to deplete intracellular glucose. At the end of each experiment, the cells were washed twice with PBS, and their viability was determined by the fluorescence of Calcein (200 nM) in a plate reader (Spectra Max M3, Molecular Devices, San Jose, CA, USA). Fluorescence was read at excitation of 492 nm and emission of 518 nm in endpoint mode with an integration time of 1 s. OCR and ECAR values were normalized to cell viability.

### 3.6. 2-DG Treatment and Cell Viability by Calcein-AM Assay

BCP-ALL cells (2.5 × 10^5^) were starved for 4 h in serum-free medium (RPMI1640 for cell lines and AIM-V for primary cells). After starvation, cells were treated with rIGF1 (50 ng/mL), insulin (3 nM, Novo Nordisk, Bagsvaerd, Denmark), and/or rIGFBP7 (100 ng/mL) for 4 h. Where indicated, cells were pretreated with an anti-IGFBP7 antibody (clone C311, 20 µg/mL) or Ly294002 (30 µM), which was added 30 min before rIGF1+rIGFBP7 or insulin+rIGFBP7 treatment initiation. 2-DG (40 mM, Sigma-Aldrich, St. Louis, MO, USA) treatment started after 4 h of rIGF1, insulin, and/or rIGFBP7 stimulation. Cellular viability was measured by Calcein-AM (Thermo Fisher Scientific, Waltham, MA, USA). Calcein positive cells were monitored in a timecourse by flow cytometry using a LSR Fortessa cytometer (Becton Dickinson, Franklin Lakes, NJ, USA) 30 min after addition of Calcein-AM (200 nM). Data were analyzed with FlowJo software Version 10 (Becton Dickinson, Franklin Lakes, NJ, USA).

### 3.7. GLUT1 Cell Surface Expression by Flow Cytometry

BCP-ALL cells (2.5 × 10^5^) were starved in serum-free medium (RPMI1640 for cell lines and AIM-V for primary cells) for 4 h and then stimulated with rIGF1+rIGFBP7 (50 ng/mL and 100 ng/mL, respectively) for 24 h. Where indicated, cells were pretreated with an anti-IGFBP7 antibody (clone C311, 20 µg/mL) or Ly294002 (30 µM), which was added 30 min before rIGF1+rIGFBP7 treatment initiation. Cells were washed with PBS and fixed with 4% paraformaldehyde without permeabilization. Surface expression of GLUT1 was analyzed by labeling cells with the anti-hGLUT1-PE (NB110-39113PE, Novus Biologicals, Centennial, CO, USA) antibody or the corresponding isotype control (mIgG1k-PE, clone MOPC-21, Becton Dickinson, Franklin Lakes, NJ, USA) diluted in 0.5% BSA in PBS for 30 min at 4 °C. Cells were analyzed in a LSR Fortessa cytometer and with FlowJo Software Version 10 (Becton Dickinson, Franklin Lakes, NJ, USA).

### 3.8. GLUT1 Cell Surface Expression by Confocal Analysis

BCP-ALL primary cells (5 × 10^5^) were cultured in a 12-well Millicell glass camber (Sigma-Aldrich, Merck) previously treated with poly-D-lysine (100 µg/mL) and then submitted to centrifugation at 300× *g* for 5 min at room temperature to attach the cells to the wells’ surface. These cells were starved in serum-free medium (AIM-V) for 4 h and then were stimulated with rIGF1+rIGFBP7 (50 ng/mL and 100 ng/mL, respectively) for 24 h. Cells were washed 3 times with PBS, fixed with 4% paraformaldehyde without permeabilization, incubated with the unconjugated monoclonal rabbit anti-GLUT1 antibody (1:100—clone D3J3A, #12939 Cell Signaling Technology) overnight at 4 °C, washed 3 times in PBS, and incubated with anti-Rabbit IgG Fab2 Alexa Fluor (R) 555 (1:500—#4413 Cell Signaling Technology) for 1 h at room temperature. Nuclear staining was completed with DAPI solution (1 μg/mL, Thermo Fisher Scientific) for 10 min. Slides were analyzed using a Zeiss LSM 800 Confocal microscope (63× immersion), and pixel quantification was performed using Image J Fiji Software (Version 1.53t) (Becton Dickinson, Franklin Lakes, NJ, USA) for each individual cell.

### 3.9. Kaplan–Meier Survival Curves

RNA-Seq data on SLC2A1 expression at diagnosis for a series of BCP-ALL samples with overall survival information were generated by the TARGET Initiative (phs000464), which we obtained through cBioPortal (http://www.cbioportal.org (accessed on 15 February 2023)), an open-access database for Cancer Genomics [40]. Patients were divided into two groups according to the median *SLC2A1* expression: low (*n* = 40) and high (*n* = 41). Survival curves were generated by the Kaplan–Meier method and compared by the log-rank test using GraphPad Prism 8.0 software. 

## Data Availability

The data presented in this study are available on request from the corresponding author.

## Supplementary Materials

The following supporting information can be downloaded at https://www.mdpi.com/article/10.3390/ijms24119679/s1.
