# Supplementary material for: IGFBP7 Fuels the Glycolytic Metabolism in B-Cell Precursor Acute Lymphoblastic Leukemia by Sustaining Activation of the IGF1R–Akt–GLUT1 Axis
Source: Int J Mol Sci. 2023 Jun 2;24(11):9679. doi: 10.3390/ijms24119679 (PMC10253689; doi:10.3390/ijms24119679)
Supplement: Supplementary file 1 [file ijms-24-09679-s001.zip › ijms-2357332-supplementary.pdf]

## Supplementary Figure S1

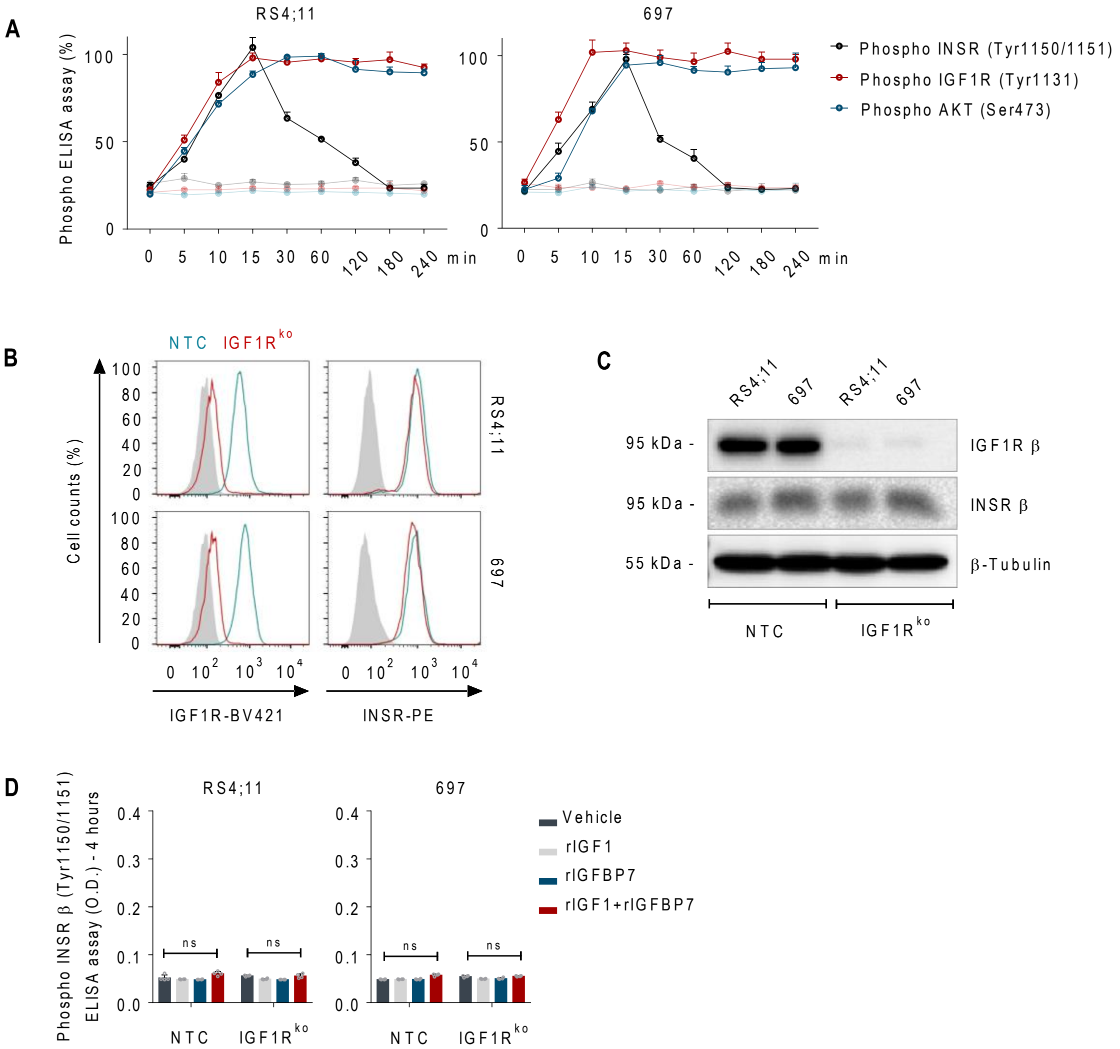


**Figure S1.** Time course of IGF1R/Akt phosphorylation and generation of IGF1R knockout BCP-ALL cell lines. (A) Normalized ELISA results for phospho-INSRβ (Tyr1150/1151), phospho-IGF1Rβ (Tyr1131) and phospho-Akt (Ser473) in RS4;11 and 697 BCP-ALL cell lines. Time-lapse experiments were performed after treatment with rIGF1+rIGFBP7 (50 and 100 ng/mL, respectively) or vehicle (light lines). Curves represent means ± SEM for two independent experiments. (B) IGF1R (left) or INSR (right) surface expression measured by flow cytometry in RS4;11 and 697 BCP-ALL cell lines (NTC, no target control and IGF1R knockout) 48 h after doxycycline activation of the CRISPR-mediated knockout. Gray histograms represents cells staining with respective isotype control antibody. (C) IGF1R, INSR and β-tubulin expression in total cell lysates measured by western blot in RS4;11 and 697 BCP-ALL cell lines (NTC, no target control and IGF1R knockout) 48 h after doxycycline activation of CRISPR knockout. Western blot protocol has been previously described.1 Briefly, membranes were immunoblotted overnight at 4 °C with anti-Insulin Receptor β (clone L55B10, #3020 Cell Signaling Technology), anti-IGF1 Receptor β (clone D23H3, #9750 Cell Signaling Technology) and β-tubulin (clone 9F3 #2128 Cell Signaling Technology). Images were acquired with a ChemiDoc equipment (Bio-Rad). (D) ELISA results for phospho-INSRβ (Tyr1150/1151) in RS4;11 and 697 BCP-ALL cell lines (NTC, no target control and IGF1R knockout) after 4 h of treatment with rIGF1 (50 ng/mL) and/or rIGFBP7 (100 ng/mL). Bars represent means ± SEM for four independents experiments; ns = not significant.

## Supplementary Figure S2

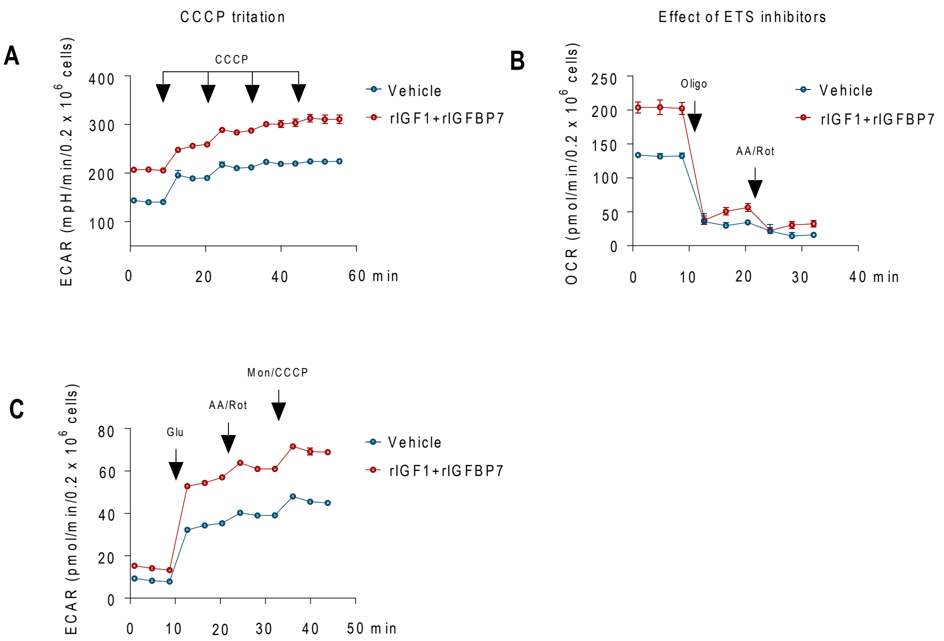


**Figure S2.** (**A-B**) One representative oxygen consumption rate (OCR) traces in RS4;11 cell line after 4 h of treatment with rIGF1+rIGFBP7 (50 ng/mL and 100 ng/mL, respectively) or control (vehicle). (**A**) Arrows indicate sequential injections of CCCP (total amount of 1.2 µM) to reach maximal OCR. (**B**) Arrows indicate oligomycin (Oligo, 1 µg/mL) and antimycin+rotenone (AA/Rot, 1 µM each) injections to evaluate fractions of OCR linked to ATP synthesis and non-mitochondrial OCR, respectively. (**C**) One representative extracellular acidification rate (ECAR) traces in RS4;11 cell line after 4 h of treatment with rIGF1+rIGFBP7 (50 ng/mL and 100 ng/mL, respectively) or control (vehicle). Arrows indicate glucose (Glu, 10 mM), antimycin+rotenone (AA/Rot, 1 µM each) and monensin+CCCP (Mon, 200 µM; CCCP, 1 µM) injections.

## Supplementary Figure S3

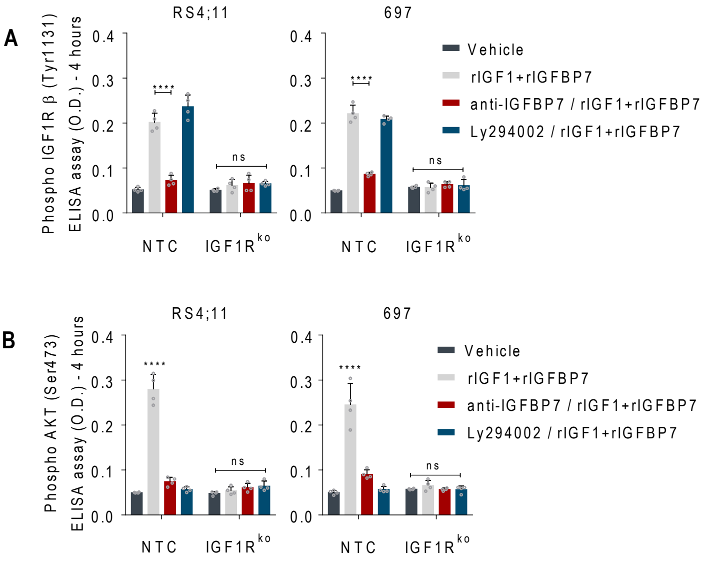


**Figure S3.** Sustained IGF1R/Akt phosphorylation in normal and IGF1R knockout BCP-ALL cells. ELISA results for (A) phospho-IGF1Rβ (Tyr1131) and (B) phospho-Akt (Ser473) in RS4;11 and 697 BCP-ALL cell lines (NTC, no target control and IGF1R knockout) 4 h after rIGF1+ rIGFBP7 treatment (50 and 100 ng/mL, respectively). Where indicated, cells were pretreated with an anti-IGFBP7 antibody (clone C311, 20 µg/mL) or Ly294002 (30 µM) which were added 30 min before rIGF1+rIGFBP7. Bars represent means ± SEM for four wells. Statistical analyses were done by 2-way ANOVA and Bonferroni posttests (****P≤0.0001); ns = not significant.

## Supplementary Figure S4

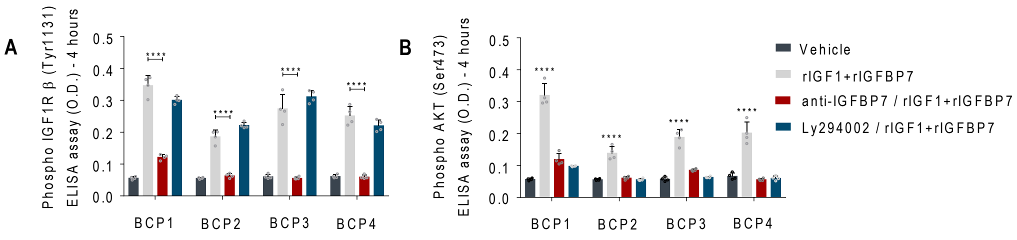


**Figure S4.** Sustained IGF1R/Akt phosphorylation in primary BCP-ALL cells. ELISA results for (A) phospho-IGF1Rβ (Tyr1131) and (B) phospho-Akt (Ser473) in four different primary BCP-ALL samples 4 h after treatment with rIGF1+rIGFBP7 (50 and 100 ng/mL, respectively) or vehicle. Where indicated, cells were also treated with an anti-IGFBP7 antibody (clone C311, 20 µg/mL) or Ly294002 (30 µM) which were added 30 min before rIGF1+rIGFBP7. Bars represent means ± SEM for four wells. Statistical analyses were done by 2-way ANOVA and Bonferroni posttests (****P≤0.0001).

**Supplementary Figure S5**

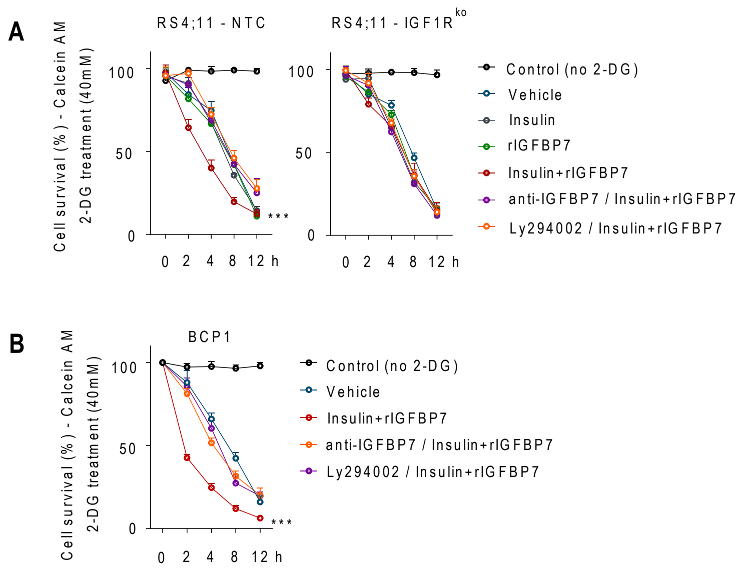


**Figure S5.** Calcein AM cell viability assays on BCP-ALL cells exposed to lethal doses of 2-deoxy-D-glucose (2-DG). Cell viability results for the (**A**) RS4;11 BCP-ALL cell line (NTC: no target control and IGF1R knockout) and (**B**) BCP1 primary BCP-ALL sample. For all conditions (except for the "no 2-DG" control group, black lines) cells were pretreated with rIGF1 (50 ng/mL) and/or rIGFBP7 (100 ng/mL) for 4 h and then subjected to 2-DG treatment (40 mM) for up to 12 h. Where indicated, cells were also pretreated with an anti-IGFBP7 antibody (20 µg/mL) or Ly294002 (30 µM) which were added 30 min before rIGF1+rIGFBP7. Viable cells (Calcein+) were determined by flow cytometry at each indicated time point. Curves represent means ± SEM for three independents experiments. Statistical analyses correspond to differences between areas under the curves (AUC) (***P≤0.001).

**Supplementary Table S1**

**Table S1. Clinical and biological features of primary BCP-ALL samples used in the functional studies.**

| ID | Gender | Age | WBC per $mm^3$ | CALLA | Karyotype | Follow up (years) | Status at last follow up |
|---|---|---|---|---|---|---|---|
| **BCP1** | M | 9.06 | 4,230 | Pos | 57, XXYY, +4, +6, +8, +10, +15, +17, +18, +21, +21 | 6.732 | Alive |
| **BCP2** | M | 15.33 | 37,690 | Pos | 49, XXY, +17, +22 | 3.789 | Death |
| **BCP3** | M | 4.10 | 34,450 | Pos | 54, XXY, +6, +14, +17, +18, +21, +22 | 6.590 | Alive |
| **BCP4** | F | 3.78 | 6,800 | Pos | 58,XX, der(4) t(1;4) (p13;16), +4,+5,+6,+9,+10,+12,+14,+17,+18,+19 | 5.347 | Alive |

ID: patient's identification; F: female; M: male; Age: age in years; Pos: positive (>20%); WBC: withe blood cells; CALLA: common acute lymphoblastic leukemia antigen (CD10).
